# Detection of Atmospheric Methyl Mercaptan Using Wavelength Modulation Spectroscopy with Multicomponent Spectral Fitting

**DOI:** 10.3390/s17020379

**Published:** 2017-02-16

**Authors:** Zhenhui Du, Jiaxin Wan, Jinyi Li, Gang Luo, Hong Gao, Yiwen Ma

**Affiliations:** 1 State Key Laboratory of Precision Measuring Technology and Instruments, Tianjin University, Tianjin 300072, China; Melissaslove@sina.com (J.W.); luogang@tju.edu.cn (G.L.); gaohongtju@tju.edu.cn (H.G.); yiwenma@tju.edu.cn (Y.M.); 2 Key Laboratory of Advanced Electrical Engineering and Energy Technology, Tianjin Polytechnic University, Tianjin 300387, China; lijinyi@tjpu.edu.cn

**Keywords:** methyl mercaptan sensor, distributed feedback interband cascade laser (DFB-ICL), multicomponent spectral fitting, wavelength modulation spectroscopy (WMS), mid-infrared spectroscopy

## Abstract

Detection of methyl mercaptan (CH_3_SH) is essential for environmental atmosphere assessment and exhaled-breath analysis. This paper presents a sensitive CH_3_SH sensor based on wavelength modulation spectroscopy (WMS) with a mid-infrared distributed feedback interband cascade laser (DFB-ICL). Multicomponent spectral fitting was used not only to enhance the sensitivity of the sensor but also to determine the concentration of interferents (atmospheric water and methane). The results showed that the uncertainties in the measurement of CH_3_SH, H_2_O, and CH_4_ were less than 1.2%, 1.7% and 2.0%, respectively, with an integration time of 10 s. The CH_3_SH detection limit was as low as 7.1 ppb with an integration time of 295 s. Overall, the reported sensor, boasting the merits of high sensitivity, can be used for atmospheric methyl mercaptan detection, as well as multiple components detection of methyl mercaptan, water, and methane, simultaneously.

## 1. Introduction

Methyl mercaptan (methanethiol, CH_3_SH, MM), a colorless gas with a foul odor, is produced by both natural [1] and industrial sources [2]. The olfactory threshold of MM is very low (~2 ppb). It is significantly toxic at high concentrations and can destroy the central nervous system of living beings. The recommended threshold concentration limit of MM, as defined by the American Conference of Governmental Industrial Hygienists (ACGIH), is 0.5 ppm (threshold limit value: 8 h time weighted average; TLV-TWA) [3]. CH_3_SH is also a predominant marker in exhaled breath (ppm levels) for the diagnosis of periodontal and stomach diseases [4,5]. Therefore, it is necessary to monitor CH_3_SH continuously from ppb to ppm levels for purposes of environmental monitoring and biomedical diagnosis.

Several common methods are used for CH_3_SH detection, including combined gas chromatography and mass spectroscopy (GC–MS) [6,7], gas sensors [3,8,9], chemiluminescence [10,11,12], and mid-infrared (MIR) laser spectroscopy [13,14]. GC–MS and sensors such as biosensors can provide accurate analyses down to a few ppb; however, these methods are either costly or have a short lifetime. Electrochemical devices are only sensitive to ppm level of CH_3_SH, which greatly exceeds the olfactory threshold [8]. Gaseous CH_3_SH should be collected and react with certain chemical substances before the detection using chemiluminescence. Although it can achieve ppb levels and near real-time detection, it is complex for the maintenance of the equipment. MIR laser spectroscopy has also been used for CH_3_SH detection due to its high sensitivity and fast response. Vance et al. [13] characterized CH_3_SH absorption using a carbon isotope laser spectrometer. A 3.27 µm infrared tunable laser spectrometer (TLAS) was recommended for monitoring CH_3_SH in situ and real time as earth-based bio-signatures for extraterrestrial exploration. The selected spectral line absorption can be influenced by significant interference, especially by methane absorption. We previously examined CH_3_SH absorption features from 3260 to 3400 nm and selected the spectral line at 3393.584 nm for measuring CH_3_SH using direct absorption TLAS technology [14]. The detection limit was 25 ppb, and the integration time was 1.84 s under laboratory conditions in the measurement of pure CH_3_SH. However, the detection of CH_3_SH in the atmosphere will be disturbed by interfering atmospheric absorption, while CH_3_SH concentration is lower than several ppm [14].

In this paper, an improved CH_3_SH sensor with high sensitivity based on wavelength modulation spectroscopy (WMS) was developed to detect atmospheric CH_3_SH using a 3392 nm MIR interband cascade laser (ICL). A multicomponent spectral fitting approach was used to eliminate the influence of the main atmospheric interferents, H_2_O and CH_4_. A series of experiments were performed to verify the performance of the sensor.

## 2. Methods

In wavelength modulation spectroscopy [15,16,17], the injection current of a tunable laser is modulated by a high-frequency sinusoid superimposed on a repetitive ramp. Therefore, the incident laser frequency (wavelength), ν(t), can be expressed as [17]

(1) $$\nu (t)=\bar{\nu }+a\cos(\omega t)$$

where $\bar{\nu }$ is the average frequency (wavelength) of the laser, and a is the amplitude of the frequency (wavelength) modulation.

According to the Beer–Lambert law, for optically thin samples, α(v)<0.1, where α(v) is the absorption coefficient at frequency ν, and the transmission coefficient τ can be written as

(2) $$\tau (v)=\left(\frac{I_{t}}{I_{0}}\right)=\exp\left[-\alpha (v)\right]=\exp(-S\phi_{v}PxL)\approx 1-\alpha (v)$$

The spectral absorbance, $\alpha (v)=\alpha \left[\bar{v}+a\cos(2\pi ft)\right]$, is an even periodic function in 2π*ft* and can be expanded as a Fourier cosine series:

(3) $$-\alpha \left[\bar{\nu }+a\cos(\omega t)\right]=\sum_{k=0}^{\infty }H_{k}\left(\bar{\nu },a\right)\cos(k\omega t)$$

Typically, the case of *k* = 2 is widely used in WMS, because the second harmonic signal (2*f*) is closely related to the absorption and is free of background. The second harmonic signal of a mixture of *N* types of gases can be expressed as

(4) $$H_{2}\left(\nu ,a\right)=-\frac{PL}{\pi }\sum_{i=0}^{n}x_{i}\sum_{j=0}^{m}\int_{-\pi }^{\pi }S_{ij}\phi_{ij}\left(\nu +a\cos\theta \right)\cos2\theta d\theta$$

where $x_{i}$ is the mole fraction of the $i_{th}$ absorbing species; $S_{ij}$ (*cm/molecule*) and $\phi_{ij}$ are the line strength and line shape function of the $j_{th}$ line of the $i_{th}$ absorbing species, respectively; *P* is the total pressure of the mixture; *L* is the path length. To simplify Equation (4), if the temperature and pressure of the gas sample remain constant, and the line shape variation is ignored, then Equation (4) can be expressed as

(5) $$H_{2}\left(\nu ,a\right)=\sum_{i=1}^{n}x_{i}*H_{2\_i\_per}$$

where $H_{2\_i\_per}$ is the second harmonic signal of the $i_{th}$ species in per unit volume concentration. Equation (5) means that the second harmonic signal of a mixture is equal to the sum of the product of each component’s concentration and that its second harmonic signal in per unit volume concentration when the condition of optically thin is met.

The magnitude of the absorption-based *2f* signal in Equation (4), $S_{2f}(\bar{v})$, which is usually measured by a lock-in amplifier, can be described as

(6) $$S_{2f}(v)\approx \frac{GI_{0}}{2}\cdot H_{2}\left(\nu ,a\right)=\sum_{i=0}^{n}x_{i}*S_{2f\_i\_per}$$

where G is the electro-optical gain of the detection system, and $S_{2f\_i\_per}$ is the *2f* signal of the $i_{th}$ species in per unit volume concentration, which can be measured off-line and stored in the internal memory. Therefore, $S_{2f}(v)$ of a mixture theoretically equals the sum of the product of each component’s concentration and its *2f* signal in per unit volume concentration.

**Figure 1:**
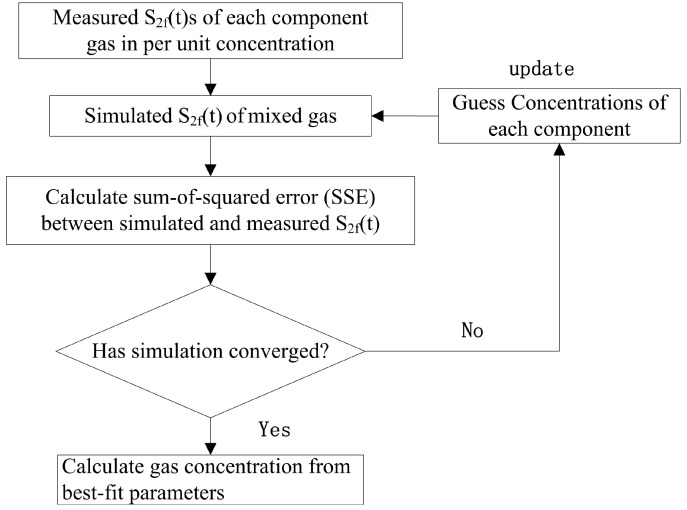
Flow chart for illustrating the multicomponent spectral fitting routine.

A multicomponent spectral fitting algorithm, based on multivariate regression and nonlinear least square fitting [16], is used to calculate the multi-component concentration in a mixture according to Equation (6). The steps of this algorithm are displayed in Figure 1.

The Levenburg–Marquardt algorithm [15] was applied during the iterative process of the method. To complete the algorithm, the reference *2f* signal of every component was obtained beforehand; the concentrations of all components in the mixture under study were non-negative parameters according to the Levenburg–Marquardt algorithm, and their initial values were given first. In each iteration of the fitting routine, the *2f* signal of the mixture was simulated according to Equation (6) with the updated parameters. The sum-of-square (SSE) between the measured and simulated *2f* was calculated to quantify the accuracy of the latter. When the SSE was minimized, the fitting routine converged upon a unique solution. Once the routine converged, the best fitting parameters were determined as the concentrations of the components.

**Figure 2:**
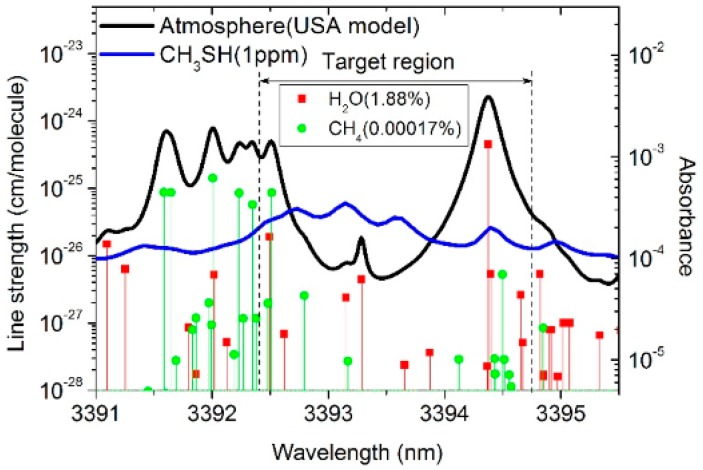
Absorption spectrum and line strength of the USA atmospheric model (mean latitude, summer, H = 0, from the HITRAN database) versus absorption spectrum of CH_3_SH at 1 ppm (from the PNNL database) and in 3391–3395.5 nm.

Based on the absorption of CH_3_SH in the MIR band [14] and the available operating wavelength range of the ICL in our laboratory, an absorption range of 3392.4–3394.7 nm was used to measure the CH_3_SH concentration. The absorption of CH_3_SH is the band spectrum with the interference of H_2_O and CH_4_ in the atmosphere (Figure 2) [18,19]. The algorithm proposed above could be applied to the detection of atmospheric CH_3_SH to eliminate the interference of H_2_O and CH_4_.

## 3. Experimental Setup

**Figure 3:**
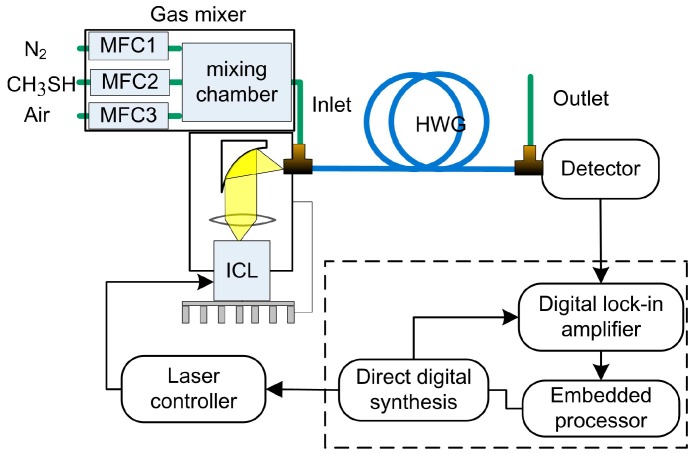
Experimental setup (MFC: mass flow controller; ICL: interband cascade laser; HWG: hollow waveguide).

The sensor mainly comprised a 3392 nm ICL (Nanoplus GmbH, Gerburnn, Germany) as the optical source, a hollow waveguide (HWG, Polymicro Technologies, Brookfield, Illinois, USA, Type HWEA10001600) as the gas cell, a laser controller (ILX Lightwave, Irvine, CA, USA, LDC-3908), a photodetector (Thorlabs, Newton, NJ, USA, PDA20H-EC), and a homemade digital lock-in amplifier (DLIA) (Figure 3). The DLIA contained a direct digital synthesizer, a phase sensitive detector, a digital low-pass filter with selectable order and time constant, and an embedded processor. The laser was placed in a homemade laser mount, and both the temperature and the current of the laser were controlled by the laser controller. The beam emitted from the ICL was collimated with an aspheric lens, coupled into the HWG by an off-axis parabolic mirror, and then collected by the photodetector. The *2f* signal was demodulated and processed by the DLIA to obtain the target gas concentration.

The dynamic spectral characteristics of ICLs have been reported to be suitable for precision spectroscopic measurements [20]. The effective optical path length of the HWG was 5 m and its volume was as small as ~4.7 mL. The injection voltage provided by the direct digital synthesizer in the DLIA was composed of a 10 Hz sawtooth ramp summed in an adder with a 2.56 kHz sine wave to obtain wavelength modulation. A period of the sine wave was made up of 256 sample points, which was determined by the direct digital synthesizer in the DLIA. The frequency of the sine wave should be an integral multiple of 256 to ensure the phase synchronization of the sawtooth and sine wave. Considering the 10 kHz bandwidth of the photodetector, a 2.56 kHz saw wave was chosen. Because of the band absorption of CH_3_SH, the modulation depth cannot be chosen accurately. After the pre-experiment was carried out, the amplitude of the sine wave was set at 25 mV, corresponding to the modulation depth of 0.15 nm [21], while the amplitude of CH_3_SH *2f* signal peak reached its maximum. According to the pre-experiment, 18 dB/oct and a 10 ms time constant of the digital low-pass filter in the DLIA were selected. The average of ten *2f* signal readings was used to determine the concentration corresponding to the temporal resolution of 1 s.

## 4. Results and Discussion

**Figure 4:**
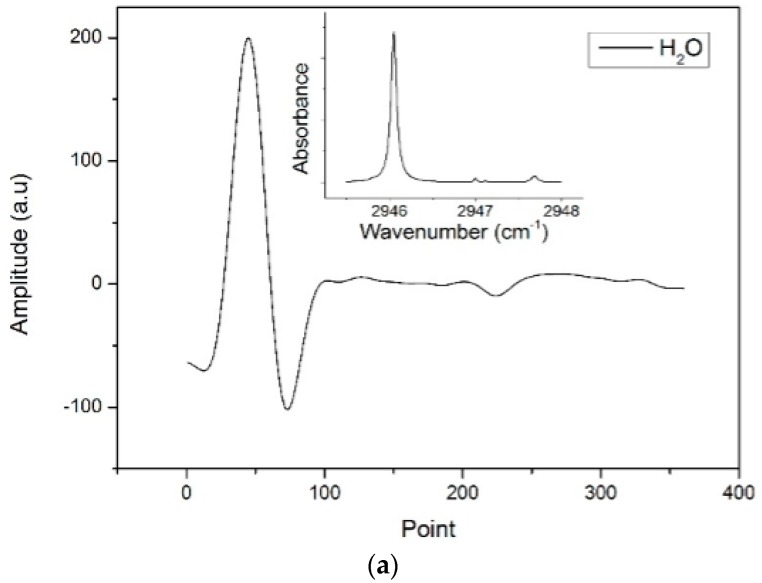

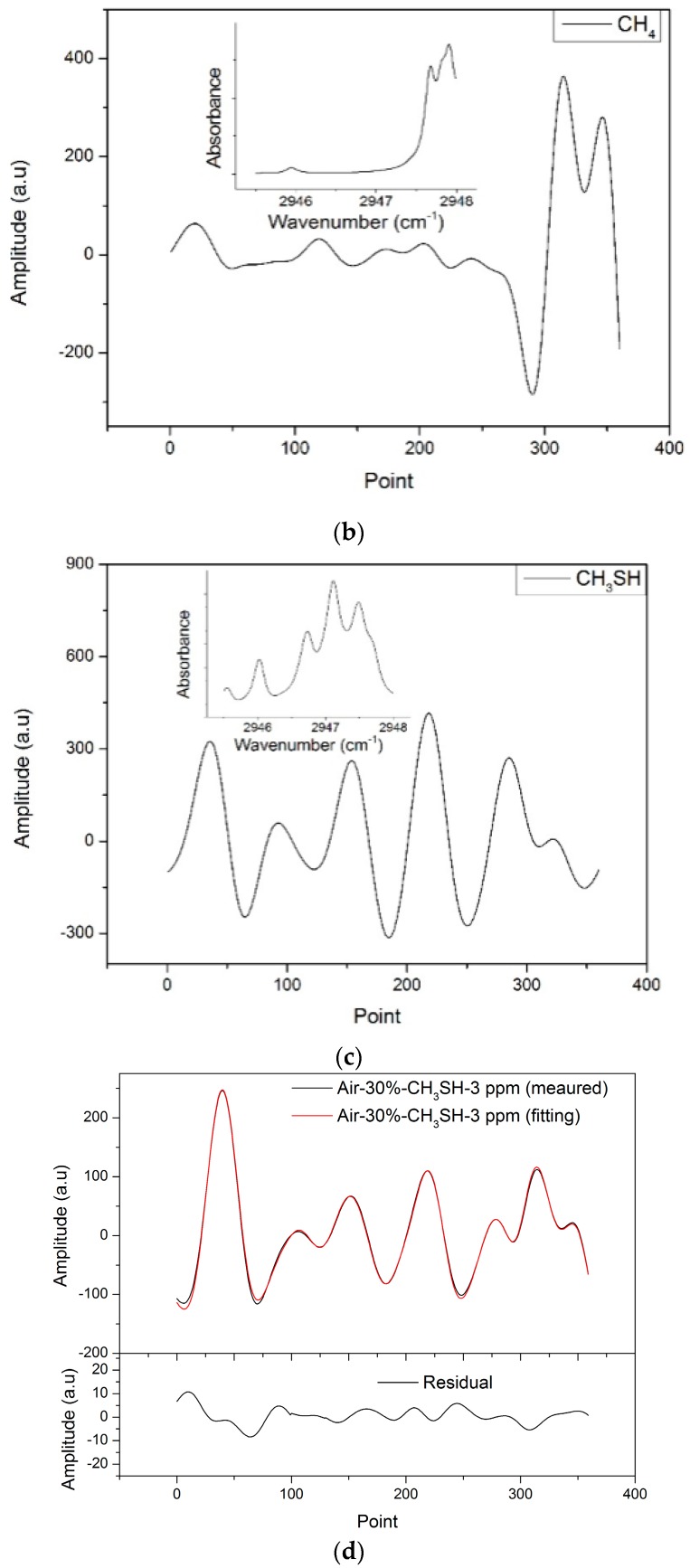
(**a**) The measured *2f* reference signal of H_2_O. The inset is the absorption spectrum of H_2_O in 2945.5–2948 cm^−1^; (**b**) The measured *2f* reference signal of CH_4_. The inset is the absorption spectrum of CH_4_ in 2945.5–2948 cm^−1^; (**c**) The measured *2f* reference signal of CH_3_SH. The inset is the absorption spectrum of CH_3_SH in 2945.5–2948 cm^−1^; (**d**) The measured and simulated *2f* signals of the mixture (air-30%-CH_3_SH-3 ppm) and the residual between them.

To obtain the reference signals for fitting according to the algorithm discussed in Section 2, the *2f* signals of CH_3_SH, CH_4_ and H_2_O were measured with the experimental system (Figure 3). The flow rate of all gas samples was controlled by a mass flow controller (MFC, Type 8715, Burkert Inc., Shanghai, China) and set to 500 mL/min. The HWG was first filled with N_2_ and then with a reference gas. Because the HWG volume was small (~4.7 mL), the reference gas immediately filled the HWG within 1 s and the etalon fringes were unable to change. Therefore, the differences between the *2f* signals of the reference gases and N_2_ could be used as reference signals that were free of etalon fringes. The reference signals of CH_3_SH and CH_4_ were obtained using reference gases with concentrations of 10 ppm and 3 ppm, respectively. In contrast, the reference signal of H_2_O was obtained using air with different concentrations of H_2_O. The concentration of H_2_O in the air was increased by a humidifier, and the difference between the *2f* signals of air with high and low concentrations of H_2_O was used as the reference signal. There was no specific concentration of the obtained H_2_O reference signal; therefore, the measured concentration of H_2_O was simply a ratio of the concentration of the H_2_O reference signals rather than an absolute value. Thus, the reference signals obtained had a high signal–noise ratio (SNR), were free of etalon fringes, and had the tuning characteristics of the laser and the response characteristics of the detector. The measured reference *2f* signals and the absorption spectrums in 2945.5–2948 cm^−1^ (3392.1–3395 nm) of CH_3_SH, CH_4_ and H_2_O are shown in Figure 4a–c, respectively.

To verify the validity of the algorithm for detecting atmospheric CH_3_SH, a series of experiments with CH_3_SH–air–N_2_ mixtures were performed. The CH_3_SH–air–N_2_ mixtures under study were prepared in a homemade gas mixer. The flows of gas samples were controlled by a homemade gas mixer that consists of three mass flow controllers (MFC, Type 8715, Burkert Inc., Shanghai, China) and were set to 500 mL/min. Two groups of concentration gradient experiments were carried out, between which the gas mixture ratio of CH_3_SH–air–N_2_ varied. In one group, the CH_3_SH concentration ranged from 2 to 20 ppm (in 2 ppm intervals), while the volume of air remained at 50%. In the second group, the volume fraction of air ranged from 10% to 70% (in 20% intervals), while the CH_3_SH concentration was fixed at 3 ppm. Each mixture was measured 10 times. The peak absorptions of all the mixtures were estimated to be less than 0.05, and the optically thin condition of WMS was satisfied. Thus, there was a linearly proportional relationship between gas concentration and the *2f* signal. All experiments were carried out with flowing gas samples in room temperature.

Figure 4d shows an example of the measured *2f* signal fitted by the simulated *2f* signal. The fitting residual in the measurement of a CH_3_SH(3 ppm)–air(30%)–N_2_ mixture is addressed. The simulated *2f* signal agrees well with the measured *2f* signal. The residual is less than 5%, which mostly consists of etalon fringes and residual amplitude modulation.

### 4.1. Accuracy

**Figure 5:**
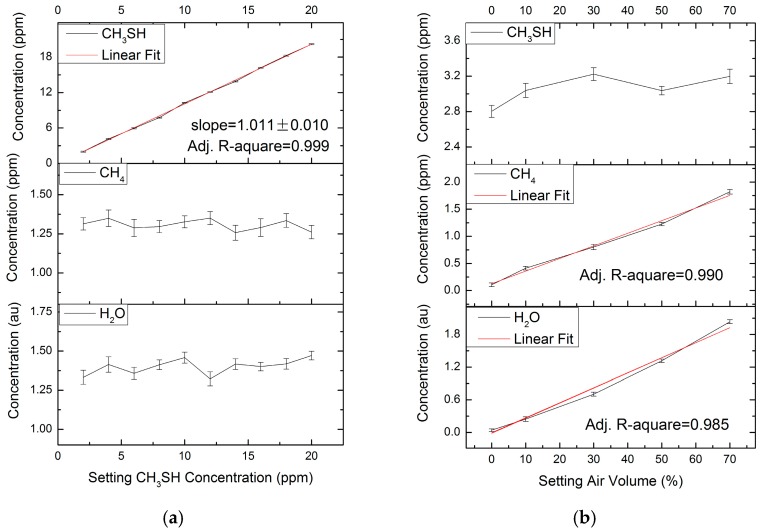
(**a**) Results of a group of mixtures with different CH_3_SH concentrations; (**b**) Results of the other group with different volume fraction of air.

The average and standard deviation of measured concentrations of CH_3_SH, CH_4_ and H_2_O in the two groups are shown in Figure 5a,b. The measured concentrations of CH_3_SH are in good agreement with the setting concentrations, with a linearity of 0.999 (Figure 5a). The accuracy of the CH_3_SH measurement is 1.1%, which can be deduced from the linear fitted slopes of 1.011 ± 0.010. The measured concentrations of H_2_O and CH_4_ are 1.307 ± 0.0954 arbitrary unit (au)and 1.401 ± 0.186 ppm, respectively. As shown in Figure 5b, the measured concentrations of H_2_O and CH_4_ agree closely with the setting volume fraction of air, with good linearity. The standard deviations of measured concentrations of CH_3_SH are no more than 0.1 ppm.

### 4.2. Uncertainty

**Figure 6:**
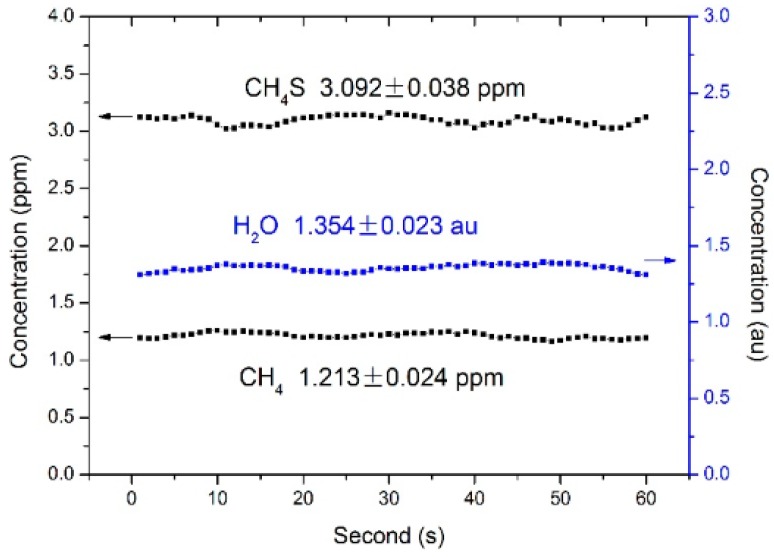
Successive measurements with 1 s intervals for a gas sample with a duration of 1 min.

Sixty successive measurements with 1 s intervals were performed with the flowing gas sample of 3 ppm CH_3_SH, 50% air fraction, and N_2_. The measurements yielded average concentration values of 3.092 ± 0.038 ppm, 1.213 ± 0.024 ppm, and 1.354 ± 0.023 au for CH_3_SH, H_2_O, and CH_4_, respectively, indicating corresponding precisions of 1.2%, 1.7% and 2.0% for the concentration measurements of the sensor (Figure 6).

The error in the concentration measurements can primarily be attributed to the uncertainties in the concentrations of CH_3_SH, CH_4_ and H_2_O during the preparation of the mixtures, while the uncertainty of the home-made gas mixer is 1.4%. Although the reference *2f* signal had a high signal–noise ratio and was free of etalon fringes, the random noise in the reference signal also influenced the uncertainties of the results, while the etalon fringes in the gas mixture *2f* signal contributed to the uncertainty as well. The uncertainties of H_2_O and CH_4_ were also caused by the concentration fluctuation of H_2_O and CH_4_ in the atmosphere.

### 4.3. Detection Limit

**Figure 7:**
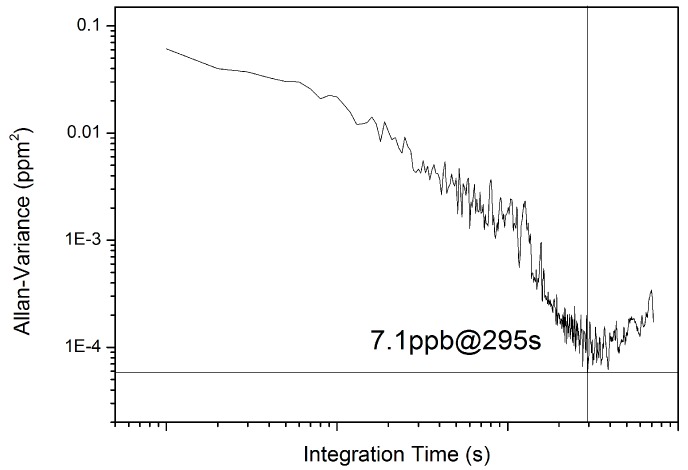
Allan variance plot for measured CH_3_SH (10 ppm).

Forty min continuous measurements of 10 ppm CH_3_SH in flow mode were performed to calculate the Allan variance. The results indicate that the sensor achieved a detection limit of 7.1 ppb with an optimal integration time of 295 s (Figure 7), which corresponds to an absorbance of 3.6 × 10^−5^. The detection limit can possibly be further improved by restraining etalon fringes and lengthening the HWG.

## 5. Conclusions

A high-sensitivity sensor based on WMS with a DFB-ICL as the optical source and an HWG as the gas cell was presented for atmospheric CH_3_SH measurement. The multicomponent spectral fitting was used to eliminate the influence of the atmospheric interferents, H_2_O and CH_4_, in CH_3_SH detection. An accuracy of 1.1%, a precision of 1.2%, and a detection limit of 7.1 ppb with an integration time of 295 s confirmed the excellent performance of the sensor for the atmospheric detection of CH_3_SH. Combined with a breath tube and a filter fixed on the gas inlet, the sensor can also be applied to CH_3_SH detection in exhaled breath for noninvasive diagnosis of relative diseases for the same interfering components with that in the air. While there are some other interferents besides H_2_O and CH_4_ that influence the detection of CH_3_SH, such as ethane [14] in the measurement environment, they can be eliminated by using the multicomponent spectral fitting introduced in this paper. Moreover, by changing the laser wavelength, the sensor can also be used with mid-infrared absorption features to detect other trace gases. As a versatile gas sensor, it can also be used for the simultaneous measurement of multicomponent gases.
